# Mass Spectrometry and Computer Simulation Predict the Interactions of AGPS and HNRNPK in Glioma

**DOI:** 10.1155/2021/6181936

**Published:** 2021-09-28

**Authors:** Wei Zhou, Ying Liu, Honglian Li, Zhaoyu Song, Ying Ma, Yu Zhu

**Affiliations:** ^1^Department of Neurology, Tianjin People's Hospital, Tianjin 300191, China; ^2^Department of Clinical Laboratory, Tianjin Huanhu Hospital, Tianjin 300350, China; ^3^Department of Medical Biochemistry and Microbiology, Biomedical Center, Uppsala University, Uppsala, Sweden; ^4^Postgraduate School of Tianjin Medical University, Tianjin 300070, China; ^5^Tianjin Key Laboratory on Technologies Enabling Development of Clinical Therapeutics and Diagnostics (Theranostics), School of Pharmacy, Tianjin Medical University, Tianjin 300070, China; ^6^Department of Clinical Laboratory, Tianjin Haihe Hospital, Tianjin 300350, China

## Abstract

Ether lipids are overexpressed in malignant tumor and play an important role in tumor process. Glioma is the most common malignant central nervous system tumor, and the content of ether lipids is higher than that of normal tissues. Alkylglycerone phosphate synthase (AGPS) is a key enzyme in the synthesis of ether esters and plays a vital role in maintaining the morphology and pathogenic properties of tumor cells. The cell proliferation and the content of tumor-related lipid such as monoalkylglycerol ether (MAGe), lysophosphatidic acid ether (LPAe), lysophosphatidylcholine ether (LPCe), lysophosphatidylethanolamine ether (LPEe), phosphatidyl inositol (PI), phosphatidylcholine (PC), and phosphatidylserine (PS) were suppressed after AGPS silencing in U251, H4, and TJ905 cells; however, heterogeneous nuclear ribonucleoprotein K (HNRNPK) could reverse the above phenomenon such as cellar proliferation and ether lipid secretion. We found that HNRNPK was the target protein of AGPS by coimmunoprecipitation and mass spectrometry assay and verified by western blot assay in U251 cells. It confirmed that AGPS and HNRNPK are coexpressed in the cellular nucleus by a confocal laser microscope. The main protein-protein interaction mechanism between AGPS and HNRNPK is hydrogen bond, conjugation bond, hydrophobic bond, and electrostatic force by computer simulation prediction.

## 1. Introduction

Glioma is the most common primary central nervous system tumor and the most common intracranial malignant tumor, and its incidence is increasing every year [1, 2]. Cancer cells metabolize lipids differently from normal cells. The content of ether lipids in tumors is higher than that in normal tissues, and the expression level of ether lipids is increased in highly invasive tumors, which is critical to the pathogenicity of cancer cells [3]. Abnormal lipid metabolism is closely related to the biological behavior of tumor cell malignant proliferation. Tumor-associated ether ester such as monoalkylglycerol (MAG), lysophosphatidic acid (LPA), lysophosphatidylcholine (LPC), and lysophosphatidylethanolamine (LPE) not only provide raw materials for tumor proliferation but also can be used as oncogenic signaling to activate tumor proliferation and invasion phenotypes [4–7].

Alkylglycerone phosphate synthase (AGPS) is the key enzyme for the synthesis of these ether esters. AGPS converts acylglycerone-3-phosphate into alkylglycerol-3-phosphate, which is a necessary step to generate ether lipids such as phosphatidic acid ether (PAe), lysophosphatidic acid ether (LPAe), and phosphatidyl inositol ether (PIe) which were heightened in multiple types of aggressive human cancer cells than less aggressive cancer and normal cells [8]. Studies have revealed that AGPS knockdown could decrease the levels of several ether lipid species, arachidonic acid, LPAe, and prostaglandins in cancer cells [9]. Our previous studies have shown that silencing AGPS expression level can reduce glioma cell proliferation, invasion, and downregulation of the above-mentioned ether ester content by the PI3K/Akt and mTOR signaling pathway, lncRNAs, and microRNAs, indicating that AGPS plays a vital role in maintaining the pathogenic characteristics of glioma cells. However, the direct target of AGPS has not been reported yet.

This study uses the combination of coimmunoprecipitation (Co-IP) technology and mass spectrometry technology to confirm the direct target heterogeneous nuclear ribonucleoprotein K (HNRNPK) of AGPS and verify the effect of HNRNPK on the biological functions of AGPS on U251, H4, and TJ905 cell proliferation and ether ester production. Protein-protein docking is based on the three-dimensional structure of two known proteins, and the near-natural structure of the complex is predicted by molecular simulation methods. At present, the three-dimensional crystal structure of the complex of HNRNPK and AGPS has not been reported. In this study, the three-dimensional crystal structure of the complex of HNRNPK and AGPS was established by ZDOCK and RDOCK technology. ZDOCK was a rigid docking algorithm based on fast Fourier transformation-related technology, which is used to search the translational and rotational space of the protein-protein system. RDOCK was an optimization process of CHARMm based on the energy [10]. We analyzed the interaction of key amino acid residues between HNRNPK and AGPS by energy scoring. Speculating the binding site of HNRNPK and AGPS and understanding HNRNPK and AGPS the combined structural features are conducive to understand their interactions, these lay a foundation for future AGPS mechanism research and can further guide the development of antiglioma-targeting AGPS.

## 2. Materials and Methods

### 2.1. Experimental Study

#### 2.1.1. Lentivirus Infection and Stable Cell Line Screening

Glioma U251, H4, and TJ905 cells were purchased from Cell Resource Center, Institute of Basic Medical Science, Chinese Academy of Medical Sciences, and School of Basic Medicine Peking Union Medical College and maintained at 37°C in a humidified atmosphere with 5% CO_2_ in DMEM medium (Corning) with 10% fetal bovine serum (Corning).

The day before lentivirus infection, 2 × 10^5^ U251, H4, and TJ905 cells were cultured on a 6-well plate overnight in DMEM medium containing 20% polybrene. AGPS shRNA lentivirus (shR-AGPS group) and control lentivirus (control group) were added. After 24 h, the cells were cultured in complete DMEM medium without polybrene for another 72 h at 37°C. Add puromycin (1 *μ*g/ml), change the screening solution every three days to continue the screening, dilute the single cell suspension to obtain monoclonal stable expression of AGPS-silenced glioma U251, H4, and TJ905 cell lines; slow virus-infected glioma U251, H4, and TJ905 cell lines were used as negative control groups. Two groups of AGPS-silenced cell lines (named shR-AGPS1 group and shR-AGPS2 group) were selected through monoclonal screening. The HNRNPK expression plasmid was transfected into AGPS-silenced U251, H4, and TJ905 cells by lip2000 to construct a HNRNPK rescue group (rescue-HNRNPK) cell line. Cells were maintained at 37°C in a humidified atmosphere with 5% CO_2_ in DMEM medium with 10% fetal bovine serum.

#### 2.1.2. Western Blot

5 × 10^6^ cells were lysed and protein was quantified by Bradford method [11]. The 50 ng protein samples were separated by 8% SDS-PAGE, transferred to PVDF membrane, and placed in 5% skim milk, which is shaken at room temperature for 2 h and placed in 5% skim milk containing AGPS antibody and IgG, respectively, and is shaken at 4°C overnight. Place the membrane in PBS solution with 0.05% Tween-20 and shake and rinse for 5 min, 4 times in total. Place the membrane in 5% skim milk containing HRP-secondary antibody for 1.5 h at room temperature. Place the membrane in PBS solution with 0.05% Tween-20 solution and shake and rinse for 5 min, 4 times in total. Place the film in Western Lightning™ Chemiluminescence Reagent for 30 seconds. Immediately put the film in the exposure box, and expose the photosensitive film in a dark room for 1 min; then, proceed with development and fixation, and use the LabWorks™ gel imaging and analysis system to take pictures.

#### 2.1.3. BrdU Assay

Cell proliferation was performed by BrdU assay as reference [12]. 3000 cells/well were seeded into a 96-well plate and cultured at 37°C for 72 h. The BrdU cell proliferation kit (Abcam) was used to determine the cell proliferation according to the manufacturer's instructions. Briefly, 20 *μ*l/well of BrdU label was added at 37°C for 12 h, cells were fixed by 200 *μ*l/well 3.7% formaldehyde in PBS at room temperature for 30 min, and 100 *μ*l/well anti-BrdU monoclonal detector antibody (1 : 2000) was added after washing for 3 times by PBS and then incubated for 1 h at room temperature. 100 *μ*l/well peroxidase-conjugated goat anti-mouse IgG antibody (1 : 2000) was added after washing for 3 times by PBS and then incubated for 30 min at room temperature. 100 *μ*l/well TMB peroxidase substrate was added after washing 3 times using PBS and incubated for 30 min at room temperature in the dark. Finally, 100 *μ*l of stop solution was added, and the optical density (OD) value was measured using the Multiskan™ Spectrum at 450 nm (Thermo Fisher Scientific, Inc.).

#### 2.1.4. Lipidomic Analysis

Targeted lipidomic analyses were performed as reference [9]. Briefly, 1 × 10^6^ cells were collected and centrifuged at 1400 g at 4°C for 10 min and extracted by 4 ml chloroform : methanol : Tris buffer (2 : 1 : 1 mixture) with inclusion of internal standards pentadecanoic acid (10 nmol) and C12:0 dodecylglycerol (10 nmol). Organic and aqueous layers were centrifuged at 1400 g at 4°C for 10 min. The organic layer was collected, and the aqueous layer was acidified by 0.1% formic acid and extracted by 2 ml chloroform. Organic layers were combined and dried by N_2_. It was dissolved in 120 *μ*l chloroform and of which 10 *μ*l was analyzed by untargeted LC-MS with a Luna reverse-phase C5 column (50 mm × 4.6 mm with 5 *μ*m diameter particles, Phenomenex). MS analysis was performed with Exactive HF LC-MS/MS (Thermo Fisher Scientific) with single-reaction monitoring (SRM).

#### 2.1.5. Co-IP Assay

5 × 10^6^ U251 cells were scraped, precooled RIPA buffer was added and slowly shaken for 15 min at 4°C, 14000 g centrifugation for 15 min, and Protein A Sepharose beads (50% concentration, per 1 ml total protein/100 *μ*l) were added, shaken for 10 min at 4°C, and centrifuged at 14000 g for 15 min at 4°C to remove the Protein A beads. The protein concentration was determined by Bradford method [11], and the total protein was diluted with PBS to about 1 *μ*g/*μ*l. Rabbit anti-AGPS antibody (6 *μ*g) and rabbit anti-IgG (6 *μ*g) were added into 500 *μ*l total protein, respectively. After being incubated overnight at 4°C, 100 *μ*l Protein A agarose beads were added to capture the antigen-antibody complex. After being incubated overnight at 4°C, centrifuged at 14000 g for 5 s, agarose bead-antigen-antibody complex was collected, supernatant removed, and washed 3 times with 800 *μ*l of precooled RIPA buffer. Suspend the agarose bead-antigen-antibody complex with 60 *μ*l loading buffer, mix gently, boil for 5 min, and use the supernatant through 8% SDS-PAGE to separate the protein by electrophoresis.

#### 2.1.6. Silver Stain Detection

The protein was separated by 8% SDS-PAGE electrophoresis, and the gel was immersed in a fixative solution (ethanol : glacial acetic acid : water 30 : 10 : 60) and shaken gently for 12 h at room temperature to fix the protein. Add 30% ethanol and shake gently for 30 min at room temperature, discard the ethanol, and repeat once; add deionized water, keep the gel at room temperature and shake gently for 10 min, and repeat twice; add 0.1% silver nitrate solution, shake the gel gently for 30 min at room temperature and avoid light, discard the silver nitrate solution, and rinse both sides of the gel with deionized water for 20 s each. Add fresh developer (2.5% sodium carbonate and 0.02% formaldehyde in water, precooled). When the stained bands of protein appear, wash the gel surface with 1% acetic acid for several minutes to stop the reaction, and then, rinse with deionized water several times, each time for 10 min. Other proteins with different molecular weights than AGPS protein appear on the gel. After cutting the gel, it is sent to mass spectrometry to screen proteins that may interact with AGPS.

#### 2.1.7. Mass Spectrometric Detection

Cut the other protein positions in the silver stain into 1 mm^3^, wash with 200 *μ*l of water twice for 10 min each time, add 200 *μ*l of 10 mM dithiothreitol (dissolved in 25 mM ammonium bicarbonate), 37°C water bath for 1 hour, cool to room temperature, blot dry, quickly add 200 *μ*l of 50 mM indole-3-acetic acid (dissolved in 25 mM ammonium bicarbonate), placed in a dark room for 45 min, washed with 25 mM ammonium bicarbonate twice and 25 mM NH_4_HCO_3_+50% acetonitrile (ACN) twice, and ACN once, each for 10 min, and vacuum dry for 10 min. Add 2 *μ*l of 0.01 *μ*g/*μ*l trypsinase solution (dissolved in 25 mM ammonium bicarbonate), leave it at 4°C for 30 min, add 25 mM ammonium bicarbonate to a total volume of 600 *μ*l, overnight at 37°C, centrifuge to collect the digestion supernatant, use extraction buffer for the remaining micelles (67% ACN, 5% formic acid (FA), and 28% water), ultrasonic for 15 min, repeat 3 times, mix the enzymolysis solution, vacuum ultradry, and carry out liquid phase (EASY-nLC 1000 System Thermo) and mass spectrometry (Q-Exactive, Thermo) according to the elution gradient parameters in Table 1. The generated mass spectrometry results were identified using software Proteome Discoverer 1.4, a supporting commercial software of Thermo Company.

#### 2.1.8. Laser Confocal Microscope Test

Inoculate 5 × 10^5^ U251 cells in a 24-well plate (place slides in advance), continue to incubate at 37°C for 72 h, wash the cells once with PBS, add 300 *μ*l 4% paraformaldehyde to fix the cells, and let stand at room temperature for 30 min. Wash the cells with precooled PBS three times, 5 min/time, add 300 *μ*l of 0.05% Triton-X-100 (diluted in PBS) to each well for permeabilization, incubate at 4°C for 5 min, add 300 *μ*l of 10% donkey serum (dilute with PBS), stand for 2 h at room temperature, aspirate the blocking solution, and add 200 *μ*l of primary antibody binding solution (anti-AGPS (mouse)/HNRNPK (rabbit) antibody) diluted with 1% donkey serum, overnight at 4°C. Take out the slides and equilibrate at room temperature for 30 min. Wash the cells with precooled PBS three times, 5 min/time. Add 200 *μ*l of 1% donkey serum-diluted secondary antibody binding solution (TRITC-labeled donkey anti-rabbit IgG secondary antibody, FITC-labeled donkey anti-mouse IgG secondary antibody) to each well, incubated for 2 h at 4°C in the dark. Wash the cells with precooled PBS three times, 5 min/time, add 200 *μ*l DAPI (diluted 1 : 1000, final concentration 1 *μ*g/ml), incubate at 4°C for 5 min, and wash the cells three times with precooled PBS, 5 min/time. Next, take out the slides and place them on the glass slides, add 5 *μ*l of fluorescent protective agent to each slide, and mount the slides with a cover glass. FITC fluorescence field excitation wavelength is 488 nm, and emission wavelength is 507 nm; DAPI maximum excitation wavelength is 364 nm, maximum emission wavelength is 454 nm, TRITC maximum excitation is 550 nm, and maximum emission wavelength is 620 nm; a confocal laser scanning microscope (FV1000, DLYMPUS) was used to observe and take pictures.

### 2.2. In Silico Study

#### 2.2.1. Homologous Modeling

**Figure 1:**
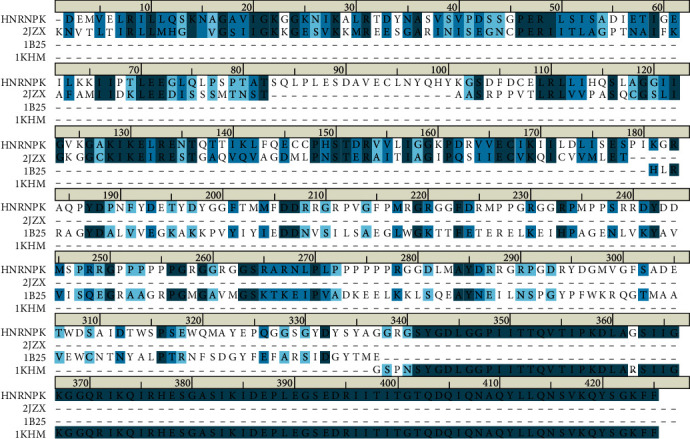
Alignment of HNRPK protein sequence with 2JZX, 1B25, and 1KHM template sequence. In order to construct the three-dimensional structure of HNRNPK protein through homology modeling technology, use the “Align Sequence to Templates” tool to align, superimpose, and calculate the sequence consistency and similarity of the target sequence with three template sequences (PDB ID is 2JZX, 1B25, and 1KHM). The deeper blue represents more consistency and similarity.

Homologous modeling was performed as reference [10]. Since the three-dimensional structure of HNRNPK protein has not been resolved, this experiment uses homologous modeling technology to construct the three-dimensional structure of HNRNPK protein. An ideal template needs to cover the length of the entire target sequence and have high sequence identity. According to the above principles, the first 3 hit sequence PDB IDs are selected as 2JZX, 1B25, and 1KHM, respectively, as templates. Use the “Align Sequence to Templates” tool to align and superimpose the target sequence with these three template sequences. The sequence alignment results are shown in Figure 1. The sequence identity is 35.5% and the similarity is 37.5%. Use Discovery Studio v3.5's MODELER program to model HNRNPK with HNRNPK amino acid (UniProt ID: P97275) as the target sequence. Briefly, download the HNRNPK sequence from the NCBI database as a template, and use the similarity search tool BLAST to search the protein database RCSB-PDB for protein structures that are more than 30% similar to the target protein and have resolved their spatial structure. Use the “Align Sequence to Templates” tool to directly align the target sequence (HNRNPK sequence) with the protein structure searched in the previous step. Use the “Build Homology Models” module in Discovery Studio v3.5 to construct the 3D structure of HNRNPK using the protein obtained from the above method as a template structure. MODELER extracts the geometric characteristics of the template and uses the PDF function to define the geometric characteristics of the protein structure, such as bond length, bond angle, and dihedral angle, to construct the 3D structure of the target sequence. Use GROMACS v4.5.5 software package and GROMOS 43a1 force field to carry out molecular dynamics research on the optimal HNRNPK model, use PME method to calculate long-range electrostatic potential, carry out molecular dynamics simulation, simulation time is 10 ns, and use Ramachandran plot and Profile-3D to evaluate the optimized structure.

#### 2.2.2. Protein-Protein Docking

Use the ZDOCK and RDOCK modules in Discovery Studio v3.5 to realize the protein-to-protein docking calculation. HNRNPK protein was selected as the docking receptor, and download the AGPS crystal structure (PDB ID: 5ADZ) from the crystal library as the docking ligand. Firstly, use the “prepare protein” in Discovery Studio v3.5 to optimize the structure of the receptor protein and the ligand protein, including removing crystal water and treating disulfide bonds, processing metal ions, and adding terminal hydrogen atoms to protein molecules. Then, use the ZDOCK module in Discovery Studio v3.5 to predict the composite structure of HNRNPK and AGPS. Set the Euler angle step of the ligand direction of rotation sampling to 6, “RMSD Cutoff” to 6.0, “Interface Cutoff” to 9.0, “Maximum Number of Clusters” to 60, and the combination mode “Top Poses” to 2000 for calculation. The ZRANK method was used to reorder the ZDOCK docking scores, and the conformations with RMSD < 3 Å were selected [13].

Protein-protein complexes are formed through noncovalent interactions between proteins, and their main components include hydrophobic interactions, hydrogen bond interactions, and electrostatic interactions. Use the Discovery Studio v3.5 “Analyze Protein Interface” module to calculate the solvent accessible surface (SAS) of HNRNPK and AGPS and analyze the key amino acid residues at the interaction interface of HNRNPK and AGPS. The “Calculate Electrostatics” module calculates the electrostatic interaction between HNRNPK and AGPS and analyzes the electrostatic potential distribution of key amino acid residues at the binding interface of HNRNPK and AGPS. The Discovery Studio v3.5 “Calculate Interaction Energy” module calculates the interaction energy between key amino acid residues at the interaction interface between HNRNPK and AGPS. The binding site of HNRNPK and AGPS was predicted by analyzing the interaction of key amino acid residues at the binding interface of HNRNPK and AGPS.

### 2.3. Statistical Analysis

SPSS version 11.0 (SPSS Inc., Chicago, IL, USA) was used for statistical analysis. Data are presented as the mean ± standard deviation. The statistical analysis was performed using analysis of variance with Tukey's post hoc test. *P* < 0.05 was considered to indicate a statistically significant difference.

## 3. Results

### 3.1. The Influence of AGPS on the Expression of HNRNPK

**Figure 2:**
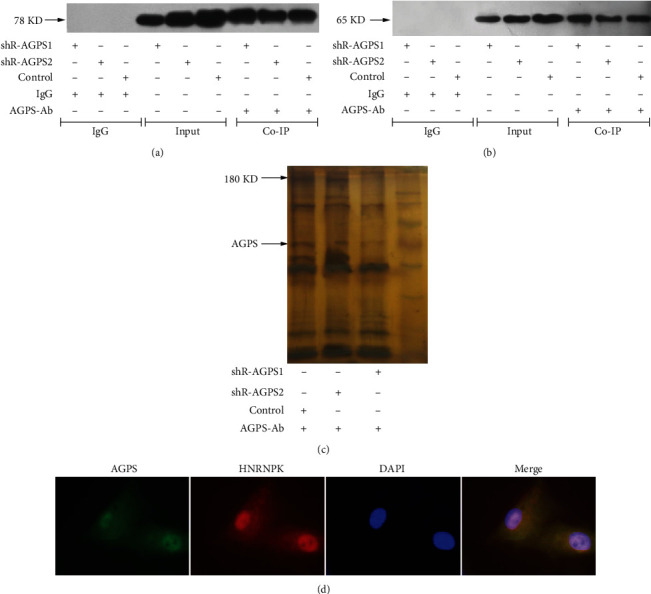
Coimmunoprecipitation of AGPS and HNRNPK in glioma cells. (a) AGPS expressed after western blot detection of coimmunoprecipitation; (b) HNRNPK expressed after western blot detection of coimmunoprecipitation; (c) silver staining result of coimmunoprecipitation; (d) localization of AGPS and HNRNPK proteins in cells.

In order to investigate the correlation between AGPS and HNRNPK, we used AGPS to perform Co-IP experiments on U251 cells that have the most significant regulation of proliferation phenotype. Compared with the control group, the expression levels of AGPS and HNRNPK in the shR-AGPS1 and shR-AGPS2 groups were significantly lower (*P* < 0.05), and the expression levels of both in the shR-AGPS1 group were obviously lower than those in the shR-AGPS2 group (*P* < 0.05) (Figures 2(a) and 2(b)), which proves that shRNA interference can significantly reduce the expression of AGPS in U251 cells, and the reduction of AGPS expression can also downregulate the expression of HNRNPK. AGPS protein and HNRNPK protein can be detected simultaneously in the cell lysate of AGPS antibody immunoprecipitation, indicating that AGPS and HNRNPK can form a complex and interact with each other.

### 3.2. Determination of AGPS Target Protein

In order to determine the target protein of AGPS, we subjected the immunoprecipitation complex in the Co-IP experiment to 8% SDS-PAGE gel electrophoresis and silver stained (Figure 2(c)). After cutting the gel nearly 180 KD, send it to mass spectrometry to screen the possible proteins that interact with AGPS.

Immunoprecipitation samples were taken for mass spectrometry. The complex of AGPS and its target protein was located in 180 KD, and there were 72 potential proteins (as shown in Table 2) bound with AGPS analyzed via mass spectrometry database score. We selected the HNRNPK by mass spectrometry database score due to the fact that HNRNPK has the highest score. Therefore, it was hypothesized that HNRNPK was the target protein of AGPS. The confocal laser microscope also confirmed that both are expressed in the nucleus (Figure 2(d)) and following study such as cell proliferation and content of tumor-related lipids.

### 3.3. The Effect of AGPS and HNRNPK on the Proliferation of Glioma Cells and the Content of Tumor-Related Lipids *In Vitro*

**Figure 3:**
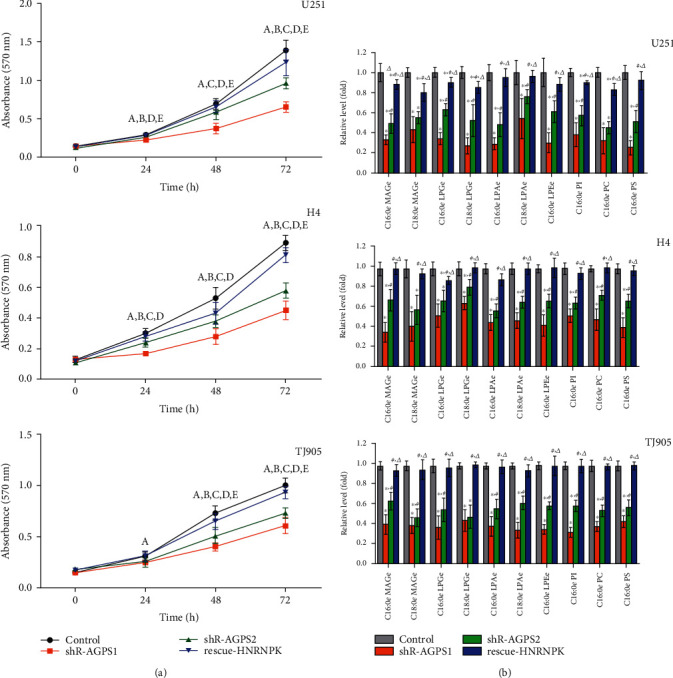
Effects of AGPS and HNRNPK on the *in vitro* proliferation of glioma cells and tumor-related lipid content. (a) After silencing the expression of AGPS in U251, H4, and TJ905 cells, compared with the control group, the cell proliferation in the shR-AGPS1 group and shR-AGPS2 group was inhibited *in vitro*. ^a^shR-AGPS1 *vs.* control group, *P* < 0.05; ^b^shR-AGPS2 *vs.* control group, *P* < 0.05; ^c^shR-AGPS2 *vs.* shR-AGPS1 group, *P* < 0.05; ^d^shR-AGPS1 *vs.* rescue-HNRNPK group, *P* < 0.05; ^e^shR-AGPS2 *vs.* rescue-HNRNPK group, *P* < 0.05. (b) The content of tumor-related lipids MAGe, LPAe, LPCe, LPEe, PI, PC, and PS was downregulated, but HNRNPK rescue can reverse the above phenotype. MAGe = monoalkylglycerol ether; LPAe = lysophosphatidic acid ether; LPCe = lysophosphatidylcholine ether; LPEe = lysophosphatidylethanolamine ether; PI = phosphatidyl inositol; PC = phosphatidylcholine; PS = phosphatidylserine. ^∗^Compared with the control group, *P* < 0.05; ^#^compared with the shR-AGPS1 group, *P* < 0.05; *^Δ^*compared with the shR-AGPS2 group, *P* < 0.05.

After silencing the expression of AGPS in U251, H4, and TJ905 cells, compared with the control group, cell proliferation in the shR-AGPS1 group and the shR-AGPS2 group was inhibited *in vitro* (Figure 3(a)). Tumor-related lipids MAGe, LPAe, LPCe, LPEe, PI, PC, and PS were downregulated, and the changes in the shR-AGPS1 group were more significant than those in the shR-AGPS2 group, but HNRNPK rescue can reverse the above phenomenon (Figure 3(b)), indicating that HNRNPK plays an important role in the regulation of glioma cell phenotype by AGPS.

### 3.4. Homologous Modeling

In this experiment, homology modeling technology was used to construct the three-dimensional structure of HNRNPK protein, and two three-dimensional structures of HNRNPK proteins were Model 1 (Probability Density Function (PDF) total energy = 3529.07, Discrete Optimized Protein Energy (DOPE) score = −32364.50) and Model 2 (PDF total energy = 3727.53, DOPE score = −31984.50). The lower the PDF total energy and DOPE score of the model are, the more reliable the quality of the model is, indicating that the model is better optimized under homologous constraints [10]. According to the above principles, Model 1 is selected as the final modeling result.

**Figure 4:**
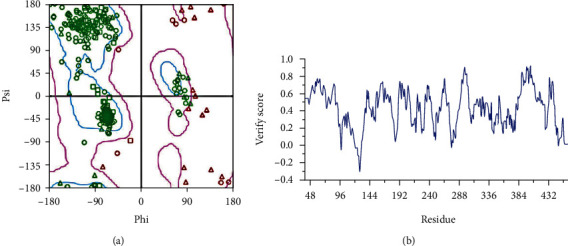
Homology modeling of the three-dimensional structure of HNRNPK protein. (a) Lagrange diagram of HNRNPK protein; (b) verify score of each amino acid residue in HNRNPK protein model.

A 10 ns molecular dynamics study was carried out on the final structure obtained by homology modeling. A frame was extracted every 100 ps, and a total of 100 conformations were selected to evaluate the rationality of the amino acid structure by Ramachandran plot to illustrate the protein or peptide stereo (Figure 4(a)). The degree of rotation (psi) of the bond between the *α* carbon atom and the carbonyl carbon atom and the degree of rotation (phi) of the bond between the *α* carbon atom and the nitrogen atom indicated the allowed and disallowed conformation in the protein or peptide amino acids.

In the Ramachandran plot, 90.31% of the amino acid residues fall in the “most suitable region,” 4.73% of the amino acid residues fall in the “general allowable region,” and 4.96% of the amino acid residues fall in the psi-phi unreasonable region of conformation, indicating that the conformation has reached the standard of the optimal model, and the skeleton structure of this protein model is reasonable.

The optimal conformation of the HNRNPK protein obtained above was evaluated using Profile-3D to evaluate the rationality of the amino acid structure. Profile-3D is a model evaluation program based on the “threading” method, which uses scoring to detect the matching relationship between the constructed model and its own amino acid sequence. The higher the score is, the greater the credibility of the homology model is [10]. The evaluation result is shown in Figure 4(b). The verify score of the modeled structure is 164.80, which is much higher than verify expected low score 87.06 and is close to verify expected high score 193.47, indicating that the protein model has a high reliability and the amino acid structures are reasonable and can be used as the starting configuration for protein-protein docking.

### 3.5. Protein-Protein Docking

#### 3.5.1. Establish the HNRNPK-AGPS Complex

**Figure 5:**
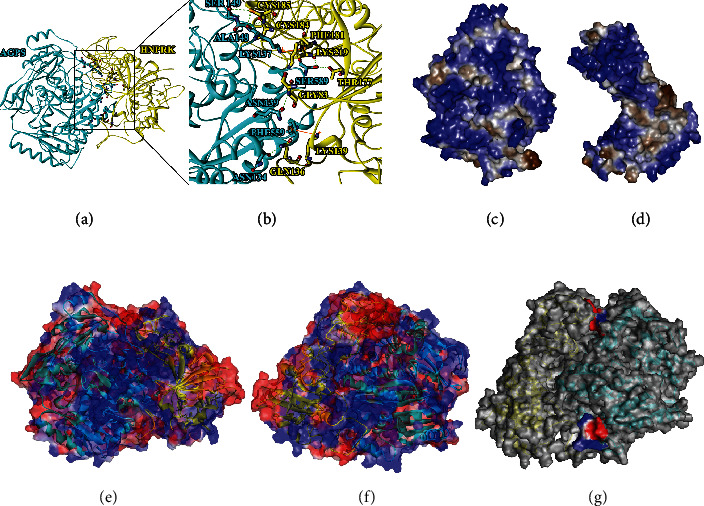
Prediction of interaction mode between AGPS and HNRNPK. (a) HNPNRK protein and AGPS protein docking diagram; (b) HNPNRK protein and AGPS protein binding interface key amino acid residue interaction diagram, yellow represents HNPNRK protein, blue represents AGPS protein, green dotted line represents hydrogen bond, and brown represents pi interaction; (c, d) hydrophobic distribution at the binding interface of the HNRPK-AGPS complex; (c) represents AGPS protein and (d) represents HNRNPK protein, blue is hydrophilic residue, and brown is hydrophobic residue; (e) the electrostatic potential distribution map of the front structure of the HNRNPK-AGPS complex; (f) the electrostatic potential distribution map of the back structure of the HNRNPK-AGPS complex; (g) the electrostatic potential distribution map at the binding interface of the HNRNPK-AGPS complex; blue is the positive potential area, red is the negative potential area; yellow represents HNRPK; and blue represents AGPS.

In this experiment, the ZDOCK and RDOCK methods were used to simulate the composite structure of HNRNPK and AGPS. A total of 60 clusters including 2000 conformations were generated by the ZDOCK algorithm and ranked by ZDOCK score, and the first 100 structures were selected for subsequent optimization. The RDOCK algorithm is used to optimize the structure of the selected complex, the ranking is performed according to the E_DOCK score, and the top five conformations with lower E_RDOCK score are selected (Table 3). The lower the E_RDOCK score is, the better the docking result of the pose is and the closer it is to the real docking conformation. After superimposing the AGPS protein in these five conformations (Nos. 1–5), the smaller the RMSD value is, the closer its conformation is to the true structure conformation. Therefore, pose 1 was selected as the target object to analyze the interaction of key amino acid residues at the binding interface of HNRNPK protein and AGPS protein and predict the binding site of HNRNPK and AGPS (Figure 5(a)).

#### 3.5.2. HNRNPK-AGPS Complex Interaction Analysis

Pose 1 obtained in the previous step is used as the target, and the “Analyze Protein Interface,” “Calculate Electrostatics,” and “Calculate Interaction Energy” modules are used to analyze the interaction between the HNRNPK-AGPS complex.

*(1) Hydrogen Bonding and Conjugation Analysis*. Hydrogen bonding and conjugation are important forces in molecular interactions and play an important role in the stability of the entire system. As is shown in Figure 5(b), the interaction interface of the HNRNPK-AGPS complex contains a total of 7 hydrogen bonds and 2 pi bonds that interact to maintain the stability of its three-dimensional structure. The benzene ring of HNRNPK:LYS139:NZ and AGPS:PHE559 forms a pi-sigma interaction; the benzene ring of HNRNPK:PHE181 and AGPS:LYS137:NZ forms a pi-cation interaction.  Table 4 lists all the amino acid residues involved in the formation of hydrogen bonds and their hydrogen bond lengths at the binding interface of HNRNPK protein and AGPS protein. They are mainly composed of the carbonyl oxygen of amino acid residues on one subunit and the amide hydrogen or sulfhydryl hydrogen of the amino acid residue on the other subunit that interact with each other. It is generally believed that hydrogen bonds with a length of less than 2.7 Å are short and strong hydrogen bonds, which are important for maintaining the stability of the three-dimensional structure of proteins [10]. The hydrogen bond lengths between the four pairs of amino acid residues at the binding interface (GLY83:HN-ASN139:OD1, CYS185:HG-LYS137:O, CYS184:SG-ALA148:HN, and LYS219:O-SER589: HG) are all less than 2.7 Å, which are short and strong hydrogen bonds, which are the main force for stabilizing the structure of the HNRNPK-AGPS complex.

*(2) Hydrophobic Interaction Analysis*. The balance between hydrophobicity and hydrophilicity is an important feature of protein structure. The hydrophobic interaction inside protein molecules determines its structural stability to a certain extent. It is an essential part of supporting the stability of protein spatial structure and has an extremely important impact on protein structure and function. Figures 5(c) and 5(d) are the distribution map of the hydrophobic amino acid residues of the HNRNPK-AGPS complex. Blue is the hydrophilic amino acid residue, and brown is the hydrophobic amino acid residue. The color intensity is positively correlated with the hydrophilicity and hydrophobicity, and the range from hydrophilic to hydrophobic is -3.0 to 3.0.  Table 5 lists the SAS values of polar residues and nonpolar residues at the interface of the HNRNPK-AGPS complex. Statistical analysis shows that the polar contact surface area values at the binding interface of HNRNPK protein and AGPS protein are 84.50 Å^2^ and 92.34 Å^2^, respectively, and the values of nonpolar contact surface area are 135.65 Å^2^ and 12.18 Å^2^, respectively. According to the formula, apply a semiempirical hydrophobicity (%) = (NONSAS1 + NONSAS2/NONSAS1 + POLSAS1 + NONSAS2 + POLSAS2) × 100%. A semiempirical method was used to quantitatively describe the hydrophobicity of the HNRNPK-AGPS complex. Among them, NONSAS and POLSAS, respectively, indicate that the solvents of the nonpolar groups and polar groups at the binding interface of the HNRPK-AGPS complex can approach the total surface area of the solvent. Subscripts 1 and 2, respectively, represent HNRPK protein and AGPS protein. Substituting the above formula to calculate the hydrophobicity between HNRPK protein and AGPS protein is 45.53%. GLN50, PRO84, LEU133, ARG148, THR177, ILE178, PHE181, GLN182, GLU183, LEU194, ILE218, PHE253, GLY260, ASP281, LEU307, PRO308, MET336, TYR361, GLU 362, PRO363, GLN364, and GLN445 amino acid residues in HNRPK and TRP93, ASP141, SER146, SER149, LEU150, ASN151, PRO152, PRO157, PHE195, LEU196, LEU197, MET246, PRO248, LEU444, LYS448, GLN462, TYR522, GLY556, VAL557, PRO560, PRO591, LEU592, GLU596, LYS628, and PHE363 the amino acid residues in AGPS exhibit hydrophobic properties, which makes a strong hydrophobic interface formed between the two proteins.

*(3) Electrostatic Interaction Analysis*. Electrostatic interaction analysis indicates that electrostatic interaction plays a huge role in maintaining the structure of biological macromolecules and the realization of biological functions. Figure 5(e) is the front of the HNRPNK-AGPS complex, AGPS is on the left and HNRPK is on the right; Figure 5(f) is the back of the HNRNPK-AGPS complex, HNRPK is on the left and AGPS is on the right. The front surface of the HNRNPK-AGPS complex has areas with equivalent positive and negative potentials, showing obvious complementarity of the positive and negative potentials. The back surface of the HNRNPK-AGPS complex still exhibits the same phenomenon after rotating 180° horizontally.  Table 6 statistically analyzes the average potential of each amino acid residue at the binding interface of the HNRNPK-AGPS complex. The interval ranges are −15.94 *KT*/*e* ~ 4.36 *KT*/*e* and −5.38 *KT*/*e* ~ 37.59 *KT*/*e*. *K* represented the Boltzmann constant, *T* represented the thermodynamic temperature (298 K), and *e* represented the unit charge. The positive potential region formed by the amino acid residues of GLN122, LEU123, PRO124, and LEU125 of HNRNPK and the negative potential region formed by the amino acid residues of LEU444, LYS448, ASP459, ASN461, and GLN462 of AGPS form electrostatic complementarity. The negative potential region formed by the amino acid residues TYR361, GLU362, PRO363, and GLN364 of HNRNPK and the positive potential region formed by the amino acid residues of LYS628, PHE636, and LEU658 of AGPS form electrostatic complementarity (Figure 5(g)). The blue shown represents the positive potential region, and the red represents the negative potential region. The magnitude of the electrostatic potential is positively correlated with the degree of color.

*(4) Interaction Energy Analysis*. In order to determine the important amino acid residues that play a role in the interaction between HNRNPK and AGPS, this experiment calculated the interaction energy of each amino acid residue located at the interface between HNRNPK protein and AGPS protein under the CHARMm force field. The calculation results show that the total interaction energy of HNRNPK protein and AGPS protein is -54.5593 kcal/mol, of which the van der Waals interaction energy and electrostatic interaction energy are -17.7061 kcal/mol and -42.0472 kcal/mol, respectively. The electrostatic interaction energy is significantly more negative than the van der Waals interaction energy; this shows that electrostatic interaction is the main driving force for the formation of composite structures.  Table 7 lists the interaction energy of important amino acid residues at the binding interface of HNRNPK-AGPS. The amino acid residues at the binding interface of the HNRPK protein include SER82, GLY83, GLN136, LYS139, THR177, PHE181, CYS184, CYS185, and LYS219, and the amino acid residues at the binding interface of the AGPS protein include ASN134, ASN139, LYS137, ALA148, SER149, PHE559, and SER589; their interaction can play an important role in the stability of the structure of the active region of the HNRPK-AGPS complex.

There was a pi bond and a hydrogen bond from LYS137 in AGPS, and there were three hydrogen bonds from SER589 in HNRPK; therefore, we considered that the major interaction energy was from the interaction between LYS137and SER589 due to the most pi bond and hydrogen bond.

## 4. Discussion

The infiltration of glioma cells into normal brain tissue can destroy normal brain tissue function. In the early stage, it may show irritation symptoms such as localized epilepsy, and later, it may show symptoms of neurological deficits such as paralysis, which is a sign of deterioration of glioma and the main cause of treatment failure and death [14]. Abnormally expressed lipids can regulate a series of functional genes to turn on and/or off abnormally by participating in the formation of tumor cells and signal transduction process so that tumor cells can acquire various characteristics different from normal cells and induce cell canceration and tumor proliferation, invasion, and apoptosis resistance [15, 16].

The inactivation of AGPS can reduce the expression of various lipids, such as ether lipids, prostaglandins, and acyl phospholipids, which are essential for the growth and spread of tumor cells, reducing the pathogenicity of cancer at the same time, while overexpression of AGPS can increase the survival and motility of many tumor cells (such as breast cancer 231MFP cells, melanoma C8161 cells, prostate cancer PC3 cells, and primary breast cancer cells) and promote tumor growth and invasion [17]. Therefore, AGPS may be a potential target for the diagnosis and treatment of glioma. The previous research of our group also showed that silencing the expression of AGPS can inhibit the proliferation and invasion of glioma cells *in vitro* and inhibit the expression of ether esters [18]. Therefore, we continue to study the direct targets of AGPS in this research.

HNRNPK is an oncogene, which is abnormally expressed in a variety of tumors. It has been shown to be closely related to tumor proliferation and lipid metabolism. HNRNPK plays an important role for glioma in proliferation, migration, invasion, and apoptosis in previous reports [19–21]. Silencing the expression of AGPS in glioma cells can downregulate HNRNPK, which proves the correlation between the two expressions. Through cell experiments, this study found that silencing AGPS can inhibit the *in vitro* proliferation of U251, H4, and TJ905 glioma cells and downregulate the tumor-related lipids MAGe, LPAe, LPCe, LPEe, PI, PC, and PS. Through the HNRNPK rescue experiment, after upregulating the expression of HNRNPK in the AGPS-silenced group cells, the proliferation ability of the cells *in vitro* and the cancer-promoting lipid content can be partially restored, further confirming that HNRNPK plays an important role in AGPS in regulating the proliferation and lipid synthesis of glioma.

In order to deeply explore the mechanism of AGPS and the relationship between AGPS and HNRNPK, we selected representative glioma U251 cells, hypothesized that AGPS can directly act on HNRNPK by Co-IP and mass spectrometry with the highest database score, and used confocal laser microscope to confirm that both are expressed in the nucleus, following study such as cell proliferation and content of tumor-related lipids also confirm this hypothesis. Therefore, we determined that HNRNPK is a direct target of AGPS.

Analyzing the binding mode of HNRNPK protein and AGPS protein and the amino acid residues that interact lay the foundation for the subsequent study of the interaction between HNRNPK protein and AGPS. In this experiment, the three-dimensional structure of HNRNPK protein was constructed by homology modeling technology, and two conformations of Model 1 and Model 2 were obtained. Among them, the PDF total energy and DOPE score of Model 1 were low, indicating that the quality of the model is reliable, and it can be selected as the final modeling structure. Because the obtained modeled structure may be unreasonable in space, molecular dynamics was used to optimize it, and 100 conformations were extracted to evaluate the rationality of the amino acid structure by Ramachandran plot. The optimal conformation of the HNRNPK protein was obtained, and 90.31% of the amino acid residues fell in the “optimal zone.” Further, use Profile-3D to evaluate the rationality of the amino acid structure, and the results show that the protein model has high credibility, and the amino acid structure in the protein is reasonable, which can be used as the starting configuration of protein-protein docking. The protein-protein docking method (ZDOCK and RDOCK methods) is used to simulate the interaction between the two proteins. According to the ranking of E_DOCK score, pose 1 has the lowest score, which possibly is the closest docking conformation to the real conformation. After the conformation of the AGPS protein in pose 1 and the protein in the crystal library are superimposed, the RMSD value is less than 1 Å, indicating that its conformation is close to the conformation of the real structure. By analyzing hydrogen bond interaction, conjugate interaction, hydrophobic interaction, electrostatic interaction, and interaction energy, it is found that the electrostatic interaction energy is significantly greater than the van der Waals interaction energy. The binding mode of the HNRNPK-AGPS complex and the amino acid residues that interact are predicted to be at the binding interface of HNRPK: GLY83, LYS139, THR177, PHE181, CYS184, CYS185, and LYS219, and at the binding interface of AGPS: ASN139, LYS137, ALA148, SER149, PHE559, and SER589. Among them, four pairs of amino acid residue (GLY83:HN-ASN139:OD1, CYS185:HG-LYS137:O, CYS184:SG-ALA148:HN, and LYS219:O-SER589:HG) hydrogen bond lengths are all less than 2.7 Å, which are short and strong hydrogen bonds. They are important for maintaining the stability of the three-dimensional structure of proteins. The benzene ring of HNRNPK:LYS139:NZ and AGPS:PHE559 forms a pi-sigma interaction with the benzene ring of HNRNPK:PHE181 and AGPS:LYS137:NZ to form a pi-cation interaction to maintain the stability of its three-dimensional structure. The hydrophobic ratio between HNRPK protein and AGPS protein is 45.53%, indicating that a strong hydrophobic interface is formed between the two proteins. The HNRNPK-AGPS complex exhibits an obvious complementary positive and negative potential. It can be seen that the interactions of these amino acids are mainly hydrogen bonds and conjugation interactions, indicating that hydrogen bonds and conjugation play an important role in the stability of the entire system.

The above analysis revealed the binding mode of HNRPK protein and AGPS protein and the amino acid residues that interacted, laying the foundation for the subsequent study of the interaction between HNRNPK and AGPS. However, from the *in vitro* cell proliferation and lipidomic analysis experiments, it was found that the *in vitro* proliferation of glioma cells and oncogenic ether ester expression changes caused by AGPS silencing could not be completely reversed by HNRNPK rescue, so it is possible that AGPS still exists other target genes in gliomas and needs to be explored. We think that there is a same target protein of AGPS in H4 and TJ905 cell lines; meanwhile, we think that HNRNPK may be not the sole target protein for APGS because it only partially restored the AGPS activity such as cellar proliferation and ether lipid secretion. Transcriptional regulators of HNRNPK such as brain-derived neurotrophic factor (BDNF) were upregulated in gliomas [22, 23], and we hypothesized that the AGPS-HNRNPK complex could facilitate to regulate tumor-related mRNA by BDNF, which would be explored in further study.

## Figures and Tables

**Table 1:** Gradient elution parameter.

Time (min)	A phase: 0.1% FA/water	B phase: 0.1% FA/ACN	Flow rate (nl/min)
0	95%	5%	600
16	90%	10%	600
51	78%	22%	600
71	70%	30%	600
72	5%	95%	600
78	5%	95%	600

**Table 2:** The score of AGPS target proteins in mass spectrometry database.

No.	Accession	Protein name	Gene	Score	No.	Accession	Protein name	Gene	Score
1	P61978	Heterogeneous nuclear ribonucleoprotein K	HNRNPK	40401.66	37	Q9H857	5′-Nucleotidase domain-containing protein 2	NT5DC2	383.09
2	P04264	Type II cytoskeletal 1	KRT1	15311.04	38	P16989	Y-box-binding protein 3	YBX3	337.52
3	P13645	Type I cytoskeletal 10	KRT10	13045.63	39	Q8N684	Cleavage and polyadenylation specificity factor subunit 7	CPSF7	309.30
4	P35908	Type II cytoskeletal 2	KRT2	11240.89	40	O43670	BUB3-interacting and GLEBS motif-containing protein ZNF207	ZNF207	266.77
5	P35527	Type I cytoskeletal 9	KRT9	6999.95	41	O15269	Serine palmitoyltransferase 1	SPTLC1	234.33
6	P08779	Type I cytoskeletal 16	KRT16	5528.54	42	P35637	RNA-binding protein FUS	FUS	230.69
7	P02768	Serum albumin	ALB	4813.05	43	Q14CN4	Type II cytoskeletal 72	KRT72	227.10
8	P17661	Desmin	DES	4557.83	44	P25705	ATP synthase subunit alpha, mitochondrial	ATP5A1	209.56
9	P02533	Type I cytoskeletal 14	KRT14	3605.01	45	P63104	14-3-3 Protein zeta/delta	YWHAZ	208.21
10	P02538	Type II cytoskeletal 6A	KRT6A	3559.06	46	Q96AE4	Far upstream element-binding protein 1	FUBP1	143.51
11	P13647	Type II cytoskeletal 5	KRT5	3076.56	47	Q8ND56	Protein LSM14 homolog A	LSM14A	134.51
12	Q9BQE3	Tubulin alpha-1C chain	TUBA1C	3034.20	48	P07237	Protein disulfide-isomerase	P4HB	130.87
13	Q9Y3I0	tRNA-splicing ligase RtcB homolog	RTCB	2294.13	49	P43490	Nicotinamide phosphoribosyltransferase	NAMPT	127.89
14	P13646	Type I cytoskeletal 13	KRT13	2203.34	50	P04075	Fructose-bisphosphate aldolase A	ALDOA	126.02
15	Q04695	Type I cytoskeletal 17	KRT17	2016.77	51	P14618	Pyruvate kinase PKM	PKM	97.38
16	Q13509	Tubulin beta-3 chain	TUBB3	1968.41	52	P02686	Myelin basic protein	MBP	94.43
17	P41219	Peripherin	PRPH	1886.97	53	Q9BZK7	F-box-like/WD repeat-containing protein TBL1XR1	TBL1XR1	93.94
18	P07437	Tubulin beta chain	TUBB	1858.30	54	O95470	Sphingosine-1-phosphate lyase 1	SGPL1	93.38
19	Q8NCA5	Protein FAM98A	FAM98A	1783.77	55	P81605	Dermcidin	DCD	85.59
20	P04350	Tubulin beta-4A chain	TUBB4A	1767.33	56	O94925	Glutaminase kidney isoform, mitochondrial	GLS	70.06
21	P47895	Aldehyde dehydrogenase family 1 member A3	ALDH1A3	1583.06	57	Q15392	Delta(24)-sterol reductase	DHCR24	69.37
22	Q07065	Cytoskeleton-associated protein 4	CKAP4	1229.76	58	Q9NRG9	Aladin	AAAS	65.81
23	Q13885	Tubulin beta-2A chain	TUBB2A	1104.43	59	Q92973	Transportin-1	TNPO1	63.09
24	O60506	Heterogeneous nuclear ribonucleoprotein Q	SYNCRIP	1104.23	60	Q14C86	GTPase-activating protein and VPS9 domain-containing protein 1	GAPVD1	60.81
25	Q8NC51	Plasminogen activator inhibitor 1 RNA-binding protein	SERBP1	938.68	61	P13637	Sodium/potassium-transporting ATPase subunit alpha-3	ATP1A3	50.23
26	O43175	D-3-phosphoglycerate dehydrogenase	PHGDH	933.75	62	A6NMB1	Sialic acid-binding Ig-like lectin 16	SIGLEC16	44.12
27	P60709	Cytoplasmic 1	ACTB	887.98	63	Q92945	Far upstream element-binding protein 2	KHSRP	43.48
28	Q9NSB2	Type II cuticular Hb4	KRT84	707.02	64	P06576	ATP synthase subunit beta, mitochondrial	ATP5B	33.49
29	Q9NZ09	Ubiquitin-associated protein 1	UBAP1	699.46	65	P49368	T-complex protein 1 subunit gamma	CCT3	30.70
30	O43516	WAS/WASL-interacting protein family member 1	WIPF1	692.01	66	Q9P2M7	Cingulin	CGN	29.53
31	Q15233	Non-POU domain-containing octamer-binding protein	NONO	598.08	67	P07477	Trypsin-1	PRSS1	28.61
32	P52272	Heterogeneous nuclear ribonucleoprotein M	HNRNPM	579.99	68	P02008	Hemoglobin subunit zeta	HBZ	27.12
33	Q00839	Heterogeneous nuclear ribonucleoprotein U	HNRNPU	518.19	69	Q8N9W4	Golgin subfamily A member 6-like protein 2	GOLGA6L2	25.14
34	P68104	Elongation factor 1-alpha 1	EEF1A1	506.28	70	P02042	Hemoglobin subunit delta	HBD	23.61
35	P68366	Tubulin alpha-4A chain	TUBA4A	476.37	71	Q9Y6G9	Cytoplasmic dynein 1 light intermediate chain 1	DYNC1LI1	19.57
36	Q9UBS0	Ribosomal protein S6 kinase beta-2	RPS6KB2	385.71	72	Q9NVE4	Coiled-coil domain-containing protein 87	CCDC87	0.00

**Table 3:** Scores after ZDOCK and RDOCK docking.

Receptor protein	Ligand protein	Pose no.	ZDOCK score^a^	E_RDOCK score^a^	Clash^b^
HNRPK	AGPS	1	25.32	-35.77	0
		2	24.64	-27.20	0
		3	24.10	-22.14	0
		4	23.98	-21.31	0
		5	24.18	-19.21	0

^a^Lower values of E_RDOCK and higher ZDOCK score indicate top/better docking of the complex. ^b^Clash “0” indicates no stearic clash between the proteins after being refined by the RDOCK protocol.

**Table 4:** Statistics of hydrogen bonding among residues at the binding interface of the HNRPK-AGPS complex.

HNRPK	AGPS	Distance (Å)
GLY83:HN	ASN139:OD1	2.24
THR177:HG1	SER589:OG	2.99
CYS185:HG	LYS137:O	2.22
CYS184:SG	ALA148:HN	2.39
CYS185:SG	SER149:HN	3.09
THR177:OG1	SER589:HG	3.20
LYS219:O	SER589:HG	2.21

**Table 5:** SAS values of polar residues and nonpolar residues at the interface of the HNRPK-AGPS complex.

Receptor protein (HNRPK) residue	Contact surface area	Polar contact surface area	Nonpolar contact surface area	Ligand protein (AGPS) residue	Contact surface area	Polar contact surface area	Nonpolar contact surface area
GLN50	29.08	29.08	0.00	TRP93	7.20	0.00	7.20
PRO84	2.22	0.00	2.22	ASP141	1.77	1.77	0.00
LEU133	16.34	0.00	16.34	SER146	1.27	1.27	0.00
ARG148	10.07	10.07	0.00	SER149	9.55	9.55	0.00
THR177	6.84	6.84	0.00	LEU150	13.02	0.00	13.02
ILE178	0.51	0.51	0.00	ASN151	19.17	19.17	0.00
PHE181	10.25	0.00	10.25	PRO152	8.62	8.62	0.00
GLN182	19.49	19.49	0.00	PRO157	7.76	0.00	7.76
GLU183	2.49	0.00	2.00	PHE195	6.65	0.00	6.65
LEU194	20.77	0.00	492852.00	LEU196	22.71	0.00	22.71
ILE218	1.38	0.00	0.77	LEU197	4.31	4.31	0.00
PHE253	26.59	0.00	—	MET246	6.92	0.00	6.92
GLY260	8.31	0.00	384922.00	PRO248	8.86	0.00	8.86
ASP281	0.51	0.51	6.59	LEU444	23.54	0.00	23.54
LEU307	1.11	0.00	0.31	LYS448	13.78	13.78	0.00
PRO308	15.21	15.21	0.00	GLN462	5.83	5.83	0.00
MET336	12.66	0.00	1.11	TYR522	0.83	0.00	0.83
TYR361	2.03	2.03	0.00	GLY556	7.86	7.86	0.00
GLU362	1.94	0.00	12.66	VAL557	0.25	0.25	0.00
PRO363	23.82	0.00	0.00	PRO560	9.14	0.00	9.14
GLN364	7.76	0.00	1.00	PRO591	12.74	0.00	12.74
GLN445	0.76	0.76	938892.00	LEU592	16.34	0.00	16.34
			3.82	GLU596	16.22	16.22	0.00
			0.76	LYS628	3.71	3.71	0.00
			0.00	PHE636	12.19	0.00	12.19

**Table 6:** The average potential of each amino acid residue at the binding interface of the HNRPK-AGPS complex.

Receptor protein (HNRPK) residue	Mean potential (*KT*/*e*)	Ligand protein (AGPS) residue	Mean potential (*KT*/*e*)
GLN122	-0.48	LEU444	-2.71
LEU123	-15.94	LYS448	16.91
PRO124	4.36	ASP459	-2.75
LEU125	0.83	ASN461	-5.38
TYR361	-1.32	GLN462	-2.86
GLU362	-4.65	LYS628	37.59
PRO363	-3.59	PHE636	-2.35
GLN364	2.64	LEU658	-0.26

**Table 7:** Interaction energy of residues at the binding interface of the HNRPK-AGPS complex.

Residue	Interaction energy (kcal/mol)	VDW interaction energy (kcal/mol)	Electrostatic interaction energy (kcal/mol)
AGPS:ASN134	-8.51	-1.98	-6.54
AGPS:ASN139	-5.28	-2.24	-3.05
AGPS:LYS137	-18.14	-3.48	-14.66
AGPS:ALA148	-3.69	-2.66	-1.02
AGPS:SER149	-0.78	-0.76	-0.02
AGPS:PHE559	-7.45	-3.57	-3.88
AGPS:SER589	-15.98	-3.02	-12.87

## Data Availability

All data generated or analyzed during this study are included in this published article.
